# Evaluation of Yield and Nutraceutical Traits of Orange-Fleshed Sweet Potato Storage Roots in Two Agro-Climatic Zones of Northern Ethiopia

**DOI:** 10.3390/plants12061319

**Published:** 2023-03-14

**Authors:** Gloria Peace Lamaro, Yemane Tsehaye, Atkilt Girma, Andrea Vannini, Riccardo Fedeli, Stefano Loppi

**Affiliations:** 1 Institute of Climate and Society, Mekelle University, Mekelle P.O. Box 231, Ethiopia; 2 College of Dryland Agriculture and Natural Resources, Department of Dryland Crops and Horticultural Sciences, Mekelle University, Mekelle P.O. Box 231, Ethiopia; 3 College of Dryland Agriculture and Natural Resources, Department of Land Resources Management and Environmental Protection (LaRMEP), Mekelle University, Mekelle P.O. Box 231, Ethiopia; 4 Department of Life Sciences, University of Siena, Via P.A. Mattioli 4, 53100 Siena, Italy; 5 BAT Center—Interuniversity Center for Studies on Bioinspired Agro-Environmental Technology, University of Naples Federico II, 80138 Napoli, Italy

**Keywords:** beta-carotene, diet, heritability, nutritional traits, orange-fleshed sweet potato

## Abstract

This study evaluated the genotype by environment interactions in the yield and nutraceutical traits of the orange-fleshed sweet potato (OFSP) storage root in different agro-climatic zones of northern Ethiopia. Five OFSP genotypes were cultivated at three different locations following a randomized complete block design, and the yield, dry matter, beta-carotene, flavonoids, polyphenols, soluble sugars, starch, soluble proteins, and free radical scavenging activity were measured in the storage root. The results showed consistent variations in the nutritional traits of the OFSP storage root depending on both the genotype and the location, as well as on their interaction. Ininda, Gloria, and Amelia were the genotypes that provided the higher yield and dry matter, as well as the higher content of starch and beta-carotene; they also showed a high antioxidant power. These findings suggest that the studied genotypes have the potential to alleviate vitamin A deficiency. This study demonstrated a high possibility of sweet potato production for storage root yield in arid agro-climate regions with limited production inputs. Moreover, the results suggest that it is possible to enhance the yield, dry matter content, beta-carotene, starch, and polyphenols of the OFSP storage root through genotype selection.

## 1. Introduction

The sweet potato (*Ipomoea batatas* [L.] Lam) is a major root crop plant in sub-Saharan Africa, valued for food and nutritional security [1,2,3]. Ethiopia, the second most populous country in Africa with a population of 102.4 million people and a fertility rate of 4.2 [4], faces significant challenges of malnutrition and hunger [4]. In particular, vitamin A deficiency is a persistent public health concern among children under the age of 5 and breastfeeding mothers. Unfortunately, more than half (55%) of the country’s children under the age of 5 have not been reached with supplementary high-dose vitamin A capsules, and only 5% of pregnant women have had access to iron supplements [4].

The prevalence of stunted and underweight children under 2 years old is alarmingly high, with rates of 41.1% and 36.2% in the Afar region and 39.3% and 23.0% in the Tigray region, located in the northern part of the country [5]. Moreover, Ethiopia continues to experience a burden of oxidative health disorders such as diabetes, hypertension, inflammations, HIV/AIDS, cancer, and visual impairment [4,6]. It is predicted that malnutrition and diarrhea will lead to an increase in annual deaths of 250,000 people, as projected by the World Health Organization [7]. In conclusion, Ethiopia faces significant challenges in addressing malnutrition, vitamin deficiencies, and other health issues. Urgent and concerted efforts are required to improve the nutritional status of vulnerable populations, especially children and pregnant women, and to tackle the burden of oxidative health disorders that continue to afflict the country.

Orange-fleshed sweet potato consumption reduces the prevalence of vitamin A deficiency and visual impairment among children and expectant mothers [8,9]. The adoption of improved orange-fleshed sweet potato (OFSP) genotypes rich in provitamin A by African farming communities will have a long-term positive impact on the nutrition and food security of marginalized groups [2]. This gives them the opportunity to produce and feed on sweet potatoes in considerably large amounts, helping to reduce the chance of exposure to malnutrition and the risk of cardiovascular disease; cancer, especially breast, colon, and lung ones; cataract and age-related muscular degeneration; and blindness, especially in children and pregnant and breastfeeding mothers [3,10,11,12].

The nutritional traits of sweet potatoes are of significant importance, especially for the selection of cultivars for food-based approaches to control oxidative health disorders and malnutrition [2,12,13,14]. The nutritional traits of sweet potatoes include dry matter, beta-carotene, vitamins (C, B, and E), starch, and protein, as well as mineral nutrients such as potassium, phosphorus, zinc, copper, iron, manganese, and calcium [13,14,15,16]. The most challenging work of a breeder is to combine all these traits in one genotype to meet the farmers’ and end users’ requirements [17,18].

Strong genotype-by-environment interactions (GEIs) have been reported for several of these nutritional traits [12,19,20,21]. According to Manrique and Hermann [19], the content of beta-carotene in sweet potatoes increases with higher site elevations. The content of beta-carotene in sweet potatoes is also influenced by other factors such as the cultivar type, air and soil temperature, day length, radiation, soil type, and incidence of pests and diseases, as well as agronomic practices [21,22,23]. In contrast, the dry matter content of sweet potatoes is affected by various factors, including soil quality, temperature, moisture, and mineral availability, particularly potassium, phosphorus, and nitrogen [24,25]. Moderately high temperatures between 20 and 30 °C promote a high dry matter content in sweet potatoes, which, however, begins to decline at temperatures >30 °C [26]. Most high-dry-matter-content sweet potato cultivars are white or cream-colored and do not provide the minimum daily requirement for vitamin A [26,27]. However, some orange-fleshed varieties have a high content of dry matter, as well as a content of beta-carotene high enough to meet a person’s required dietary allowance [13,14,21,28]. The majority of farmers tend to cultivate white-fleshed sweet potatoes, which a have high dry matter content, low water content, and low beta-carotene content compared with orange-fleshed sweet potato genotypes, which have higher water content and beta-carotene content but a lower dry matter content [29]. Several authors have reported fluctuations in the nutritional quality and content of cereals, such as maize, millet, rice, wheat, and barley, which have been attributed to climate change [30,31]. However, little is currently known about the nutritional quality and content of sweet potatoes in relation to climate change. Moreover, the performance of orange-fleshed sweet potato varieties has not been well documented in the Tigray and Afar regions of Ethiopia since the introduction of sweet potatoes as a food and nutritional crop.

To date, no studies have been conducted in these regions on the nutritional quality of the sweet potato genotypes that have been adopted. Furthermore, information regarding the nutritional content of orange-fleshed sweet potatoes across different agro-climatic zones is scarce. This lack of information presents a challenge for farmers in northern Ethiopia who face difficulties in selecting the most suitable sweet potato cultivar. Additionally, the upscaling of the best practices, including the introduction of new cultivars, remains a challenge. Therefore, this study investigated GEIs in the orange-fleshed sweet potato genotypes in use in northern Ethiopia for storage root yield, quality, and nutraceutical traits and heritability, across different agro-climatic zones. 

## 2. Results

The soils at the experimental sites had a high cation exchange capacity and a high content of available P and K, while the content of total N and the organic carbon were moderate; the soil pH was neutral, and the electrical conductivity (EC) was indicative of salt-free soils; and the texture was silty-loamy at site A and silty-clay at M1 and M2 (Table 1).

The results of the two-way ANOVA (Table 2) showed that variations in the genotype and location as well as their interaction were always statistically significant (*p* < 0.05) for all of the investigated nutraceutical traits. The mean values of the nutraceutical parameters are reported in Table 3. 

The Ininda genotype exhibited a significantly higher yield compared with other genotypes across all sites, while Kulfo and Melinda exhibited the lowest. A higher dry matter content was found for the genotypes Gloria, Amelia, and Ininda, with the location Aba’ala having genotypes with a higher dry matter content. The highest concentrations of beta-carotene were found at M1 and M2, with variable concentrations across genotypes. At all locations, the Ininda and Kulfo genotypes exhibited the highest total flavonoid content, whereas the pattern of the polyphenols was less distinct, with the Gloria, Ininda, and Kulfo genotypes demonstrating relatively higher values overall. The concentration of sucrose was higher than those of fructose and glucose in all genotypes; Mekelle-2 showed the highest concentrations of all soluble sugars. A high starch content was recorded in genotypes Kulfo, Gloria, and Melinda. A high protein content was found for the genotypes Amelia and Melinda, while low values were found in the genotype Gloria. The highest overall scavenging activity and reducing power of DPPH or the percentage antiradical activity (ARA %) were observed at Mekelle-1 for all genotypes. The potential of sweet potato extracts to reduce DPPH directly depended upon the concentration used (Table 4), with the best performance always observed at the concentration of 100%.

A very high broad sense heritability (H^2^) was estimated for beta-carotene; high H^2^ values were observed also for dry matter and starch, while low or very low H^2^ values were observed for all the other parameters (Table 5).

Correlations among the investigated nutritional traits are shown in Table 6. Positive correlations (r ≥ 0.50) emerged between fructose and glucose (r = 0.87), polyphenols and flavonoids (r = 0.76), polyphenols and antiradical activity (r = 0.69), sucrose and fructose (r = 0.68), sucrose and glucose (r = 0.68), sucrose and beta-carotene (r = 0.66), sucrose and yield (r = 0.57), and beta-carotene and yield (r = 0.53). Negative correlations (r ≥ −0.50) emerged between soluble proteins and starch (r = −0.72), beta-carotene and antiradical activity (r = −0.58), and beta-carotene and polyphenols (r = −0.50). 

The recommended dietary allowance (RDA)/recommended adequate intake (RAI) was high in vitamin A retinol equivalent; the highest contribution was provided by the genotype Ininda, while the lowest was provided by Kulfo (Table 7).

## 3. Discussion

There is increasing demand for cheap alternative sources of vitamin A, polyphenols, and other nutrients, especially from crop plants to be used as food-based approach in solving the problem of hunger and malnutrition in marginalized communities [32,33,34]. This study aimed to assess the response of five genotypes across three locations in terms of storage root yield and nutraceutical traits for boosting food and nutritional security in northern Ethiopia, where malnutrition rates remain high.

The storage root yield recorded in this study agrees with other studies [35,36]. The comparatively low yield at Aba’ala was expected due to its arid agro-climatic condition with limiting crop growth factors. In addition, the genotypes’ differential susceptibility to viruses may have affected the root yield of genotypes in Aba’ala as reported by other authors [35,37]. According to Lamaro et al. [37], Aba’ala is a hot spot for sweet potato viral disease. The genotypes Ininda and Gloria had high mean storage root yield, beta-carotene, and antioxidant power, which agrees with similar findings observed in Ininda, and Gloria is higher in essential macronutrients and micronutrients compared with other genotypes in the same location [38]. However, despite the arid agro-climate, the harvested yield indicates that sweet potato production is viable. The dry matter contents recorded in our study are consistent with other reports [12,36,39]. 

Although the level of beta-carotene found in our study was satisfactory, it was, however, higher than those reported in some previous studies [12,40,41,42,43]. Genotypes cultivated at M1 and M2 (semi-arid highlands) exhibited higher beta-carotene contents than those cultivated at Aba’ala (arid lowland), consistent with previous findings reported higher beta-carotene contents in OFSP genotypes from high altitudes compared with those at lower altitudes [19,44].

The high contents of starch and sucrose found in the studied genotypes are consistent with previous investigations [36,45]. A combination of high starch content and high sucrose content gives a more appealing taste to a genotype [46]. The low protein content found in our OFSP is in line with the values reported by Mohammad et al. [47] and Rodrigues et al. [48], and it was expected since it is well known that roots and tuberous roots are a very poor source of proteins.

We found a very high broad sense heritability for beta-carotene and moderately high for the content of starch and dry matter, while a low broad sense heritability was found for yield and the other traits. A very high heritability of 98% for beta-carotene content was reported by Afuape et al. [44]. In addition, Mbusa et al. [49] reported very high broad sense heritability values of 99.9% and 98.6% for beta-carotene and dry matter content, respectively, and a moderately low value of 39.9% for yield. Jones [50] reported a very low heritability estimate for storage root tuber yield content of 25% and a moderately high heritability for dry matter content of 65%, comparable to our observations. In Uganda, a moderately high broad sense heritability of 70% for dry matter content was recorded from a sweet potato diallel cross pollination [51]. Yada [12] observed high heritabilities of 90%, 70%, and 68% and a low heritability of 24% for beta-carotene, dry matter content, starch, and root tuber yield, similar to our results. Tumwegamire et al. [36] recorded heritability estimates of 94% for beta-carotene, 64% for starch, 44% for dry matter content, and 50% for yield in East African sweet potato germplasms. Todd [52] also recorded moderately high values of 77% for starch. These findings indicate the possibility of genetic improvement in the content of beta-carotene, starch, and dry matter of these genotypes through breeding.

Significant positive correlations among traits suggest that improvement in that trait could lead to significant improvement also in the other correlated traits. The positive correlations found between beta-carotene and yield, glucose, and sucrose are consistent with other studies [12,53], suggesting high chances of improvement or development of the genotypes with high dry matter content, starch, and yield, which would meet the market demand of consumers for various applications of sweet potatoes.

Consistently with Sun et al. [54] and Hannan et al. [55], genotypes with high flavonoid content correspondingly have high polyphenol content. The antioxidant activity was positively correlated with the content of polyphenols and flavonoids, as similarly observed elsewhere [54,56]. The genotypes Gloria, Kulfo, and Ininda showed the highest antioxidant properties in terms of polyphenols, flavonoids, and free radical scavenging activity. The consumption of flavonoids and polyphenols is enormously advantageous in human health, and it is well known for slowing down aging and counteracting oxidative stress, which is closely connected with health diseases such as cancer, cardiovascular disease, blindness, and diabetes [46]. Li et al. [57] recorded some breakthroughs in the antitumor effects of sweet potato proteins on human colon cancer cells and suggested their use as crop plant with anticancer properties.

Negative correlations may imply that the traits negatively correlated may be very difficult to improve together in a genotype, and negative correlations among traits have been for many years a great hindrance to the attainment of breakthroughs in the breeding of desired OFSP genotypes in sub-Saharan Africa [53,58]. High preference is given to cultivars with high (≥30%) dry matter content and tuber yield. In this study, we observed negative correlations between beta-carotene and antioxidant power and between beta-carotene and polyphenols, in line with other findings [36,59]. Several authors reported negative correlations between beta-carotene and yield and between dry matter content and starch [12,22]. Most released genotypes in East Africa have high dry matter contents and low beta-carotene, while some of them have low dry matter contents and high beta-carotene contents [12,13,36]. This study observed genotypes with acceptable dry matter contents and a reasonable beta-carotene content. The genotypes at Aba’ala (arid agro-climate) having a higher dry matter content than those in a semi-arid agro-climate may be attributed to comparatively higher mean and maximum temperatures in Aba’ala than at M1 and M2. These findings agree with another author [25], who observed variations in the dry matter content of sweet potato cultivars grown in different temperature ranges.

Beta-carotene is the main dietary source of vitamin A in orange-fleshed sweet potato storage root tubers [60], and according to the International Potato Center [61], radical prevention of vitamin A deficiency and maternal health is possible with a daily supplementation of 100–150 g of OFSP in the human diet. The genotypes Ininda, Amelia, Gloria, and Melinda had beta-carotene contents high enough to meet all the RDA/RAI for age groups ≤8 years old and more than 50% RDA/RAI for age group ≥19 years old, including pregnant women, except lactating females with 250 g (dw) intake. Tumwegamire [36] reported a 350–450% beta-carotene consumption under high intake of 250 g (fw) OFSP. The % RDA/RAI intakes observed in this study are lower than those reported by Tumwegamire [36].

## 4. Material and Methods

### 4.1. Study Area

The experiments were run at three selected sites: Aba’ala (A), Mekelle-1 (M1) and Mekelle-2 (M2). Aba’ala is located on a lowland at an altitude of 1440 m asl. The area is categorized as belonging to the arid agro-climatic zone, with an annual rainfall of 394 mm, and average minimum and maximum temperature of 18.6 °C and 34.0 °C, respectively. Mekelle-1 and Mekelle-2 are located at a highland altitude of 2220 m asl. These locations are classified as semi-arid agro-climatic zones. The annual rainfall is 406 mm for M1 and 523 mm for M2, and the average minimum and maximum temperatures are 13.1–12.2 °C and 25.3–24.4 °C for M1 and M2, respectively. Experimental plots of 11 × 17 m^2^ were randomly selected at each site.

### 4.2. Soil Analysis

Before planting, soil samples were taken diagonally across the experimental plots using a soil augur at a depth of 10–40 cm. Prior to chemical analysis, samples were air-dried, ground, and passed through a 2 mm sieve. Other samples for physical analysis were taken using a labeled metallic core. Samples were analyzed using the procedures described by Von Reeuwijk [62] and Van Ranst et al. [63].

### 4.3. Planting Material

A total of five distinct orange-fleshed sweet potato genotypes, 1 = Amelia, 2 = Gloria, 3 = Ininda, 4 = Kulfo, and 5 = Melinda, were obtained from the Tigray Agricultural Research Institute (TARI). These planting materials, except Kulfo (release-check), were from the new germplasm sourced from Mozambique for trials at TARI in Ethiopia.

The planting was carried out in the main rainy season (1–3 July 2020) at Aba’ala, Mekelle-1, and Mekelle-2 using a randomized complete block design (RCBD) with three replicates at a spacing of 0.3 × 1.0 m. The space between each plot was 0.5 m, and the space between blocks was 1 m. This spacing was uniform in all the planting sites. The plots were given numbers 1 to 15 corresponding to the genotypes in the replication blocks. Each plot was planted with 30 plants of a genotype, giving rise to 90 plants per genotype per site. The total plant population used per site was 450. The sites were ploughed twice at an interval of three weeks using a disc plough for deep seedbed preparation. Ridging followed secondary tillage three weeks later. The planting materials were cut at a length of 20 cm long with at least six nodes or more and were planted on the ridges at the onset of the main rainy season. The experiment was left to depend on the natural environmental conditions for moisture. However, since at Aba’ala, there was no rain at all at the time of planting, irrigation was provided four times (at planting and then at an interval of every four days until the 14th day after planting) to support crop establishment. No supplementary irrigation was provided at the other two sites. Weeding was performed using hand hoes for three months before the crop canopy fully covered the ground.

### 4.4. Plant Analysis

The harvesting was conducted in November 2020 starting with Aba’ala and then later Mekelle-1 and Mekelle-2. Plants were randomly collected from the middle of the experimental plots. On the storage roots, several parameters were measured to quantify the yield and nutraceutical quality. For parameters other than yield and dry matter content (DMC), the samples were freeze-dried (True-Ten Industrial Co., Taichung City, Taiwan), milled (Mini Mill, Thomas Scientific, Swedesboro, NJ, USA), and stored at −24 °C waiting for chemical analysis.

#### 4.4.1. Yield

All the storage roots, i.e., both small and damaged unmarketable tubers as well as large undamaged marketable tubers, were weighed (fresh weight), and the total storage root yield (tons per ha) was then calculated for each genotype [64].

#### 4.4.2. Dry Matter Content

About 200 g of fresh storage root samples were washed, sliced, and weighed (FWt), then dried at 65 °C for 72 h to a constant weight, and weighed (DWt) again. The percentage of dry matter was then calculated [65].

#### 4.4.3. Beta-Carotene

The beta-carotene content was determined by high-performance liquid chromatography (HPLC, Agilent 1100 system) using the separation method and mobile phase as described by Vannini et al. [66]. About 100 mg of ground samples were homogenized in 2 mL of pure ethanol (Carlo Erba, Cornaredo, Milan, Italy). Samples were centrifuged at 4000 rpm (PK110 centrifuge, Alc International S.r.l., Cologno Monzese, Milan, Italy) for 5 min and then filtered at 0.45 µm using a syringe filter (Lab Logistic Group GmbH, Meckenheim, Germany). Quantification was performed using a calibration curve of pure β-carotene (Merck, Darmstadt, Germany) in the range 5–400 μg mL^−1^. The runs were monitored at 460 nm. The results were expressed on a dry weight basis (dw).

#### 4.4.4. Flavonoids

Flavonoids were measured according to the method outlined by Heimler et al. [67]. An amount of 500 mg of ground samples was homogenized in 2 mL of 80% ethanol and then centrifuged at 15,000 rpm (Z 233 MK-2, Hermle, LaborTechnik GmbH, Wehingen, Germany) for 5 min. The supernatant (300 µL) was added to 45 µL of a 10% AlCl_3_ (Carlo Erba, Cornaredo, Milan, Italy) solution, 300 µL of a 1M NaOH (Carlo Erba, Cornaredo, Milan, Italy) solution, and 300 µL of deionized water. Samples were read at 510 nm with a UV-Vis spectrophotometer (8453, Agilent, Santa Clara, CA, USA). Quantification was performed with a calibration curve (5–200 µg mL^−1^) of quercetin (Sigma-Aldrich, Burlington, MA, USA), and the results were expressed as mg of quercetin equivalent on a dry weight basis (mg QE g^−1^ dw).

#### 4.4.5. Polyphenols

The total polyphenol content was measured following the method described by Fedeli et al. [68]. Briefly, 100 mg of ground samples were homogenized in 4 mL of 70% (*v*/*v*) acetone (Carlo Erba, Cornaredo, Milan, Italy) and then centrifuged at 4000 rpm (PK110 centrifuge, Alc International S.r.l., Cologno Monzese, Milan, Italy) for 5 min. The supernatant (0.5 mL) was added to 3 mL of deionized water, 0.125 mL of Folin–Denis reagent (Sigma-Aldrich), 0.750 mL of saturated Na_2_CO_3_ (Carlo Erba, Cornaredo, Milan, Italy), and finally 0.950 mL of deionized water. Samples were placed in an oven at 37 °C for 30 min; afterward, they were centrifuged at 4000 rpm (PK110 centrifuge, Alc International S.r.l., Cologno Monzese, Milan, Italy) for 5 min, and their absorbance was read at 765 nm using a UV-Vis spectrophotometer (8453, Agilent, Santa Clara, CA, USA). For the quantification, the absorbance of the samples was referred to a calibration curve (5–20 µg mL^−1^) of gallic acid (Sigma-Aldrich, USA) used as standard. Results were expressed as mg of gallic acid equivalent on a dry weight basis (mg GAE g^−1^ dw).

#### 4.4.6. Soluble Sugars

The soluble sugar content was measured following the method described by Fedeli et al. [69]. Ground samples of 100 mg were homogenized in 2 mL of deionized water and then centrifuged at 15,000 rpm (Z 233 MK-2, Hermle, LaborTechnik GmbH, Wehingen, Germany) for 5 min. The supernatant was filtered at 0.45 μm using a syringe filter (Lab Logistic Group GmbH, Meckenheim, Germany) and then directly analyzed by an HPLC system (600E System, Waters, Milford, MA, USA) equipped with a Waters 2410 refractive index detector. Sugar separation was allowed using deionized water as mobile phase, eluted at 0.5 mL min^−1^, and a Waters Sugar-Pak I ion-exchange column (6.5 × 300 mm) kept at 90 °C using an external temperature controller (Waters Column Heater Module, Milford, MA, USA). Quantification of sucrose, glucose, and fructose was obtained through calibration curves prepared by dissolving analytical sugars (Sigma-Aldrich, USA) in deionized water at concentrations of 0.1–20 mg mL^−1^. The results were expressed on a dry weight basis.

#### 4.4.7. Starch

The starch content was determined following the method described by Loppi et al. [70]. Briefly, ground samples of 50 mg were homogenized in 2 mL of dimethyl sulfoxide (DMSO) (Carlo Erba, Cornaredo, Milan, Italy). Then, 0.5 mL of HCl 8 M (Carlo Erba, Cornaredo, Milan, Italy) was added, and samples were placed in a ventilated oven for 30 min at 60 °C. After cooling, 0.5 mL of NaOH 8 M (Carlo Erba, Cornaredo, Milan, Italy) and 7 mL of deionized water were added. Samples were then centrifuged at 4000 rpm (PK110 centrifuge, Alc International S.r.l., Cologno Monzese, Milan, Italy) for 5 min, and 0.5 mL of supernatant was added to 2.5 mL of Lugol’s solution. After 15 min, the samples were read at 605 nm with a UV-VIS spectrophotometer (8453, Agilent, Santa Clara, CA, USA). Quantification was run using a calibration curve (10–400 μg mL^−1^) prepared with pure starch (Merck, Darmstadt, Germany). The results were expressed on a dry weight basis.

#### 4.4.8. Soluble Proteins

About 50 mg of ground samples were homogenized in 5 mL of deionized water and centrifuged at 4000 rpm (PK110 centrifuge, Alc International S.r.l., Cologno Monzese, Milan, Italy) for 5 min. Then, 0.4 mL of the supernatant was added to 1.6 mL of Bradford solution (Sigma-Aldrich, USA). The content of soluble proteins was determined using a UV-Vis spectrophotometer (8453, Agilent, Santa Clara, CA, USA), by reading the absorbance of the samples at 595 nm. Quantification was performed using a calibration curve, prepared with concentrations in the range 20–80 µg mL^−1^, of bovine serum albumin (BSA) (Sigma-Aldrich, USA) as standard. The results were expressed as mg of BSA equivalent on a dry weight basis (mg BSA eq g^−1^ dw).

#### 4.4.9. Free Radical Scavenging Activity

The free radical scavenging activity was determined following the method described by Vannini et al. [71]. About 100 mg of ground samples was homogenized in 2 mL of 80% (*v*/*v*) of ethanol and then centrifuged at 15000 rpm (Z 233 MK-2, Hermle, LaborTechnik GmbH, Wehingen, Germany) for 5 min. An aliquot of supernatant (200 µL) was added to 1 mL of 2,2-Diphenyl-1-picrylhydrazyl (DPPH) (Sigma-Aldrich, USA) solution, previously prepared by dissolving 1.85 mg of this compound in 50 mL of 80% methanol (*v*/*v*) (Carlo Erba, Cornaredo, Milan, Italy). To compare the antioxidant power of the samples, a blank and a control were prepared by adding 200 µL of 80% (*v*/*v*) ethanol (Carlo Erba, Cornaredo, Milan, Italy) in 1 mL of 80% (*v*/*v*) methanol (Carlo Erba, Cornaredo, Milan, Italy) and in 1 mL of DPPH solution, respectively. The reaction for all preparations was conducted in the dark for 1 h, and then the absorbance was read at 517 nm with a UV-Vis spectrophotometer (8453, Agilent, Santa Clara, CA, USA). The results were expressed as a percentage of antiradical activity (ARA %), according to the following formula:

$$ARA\%\;=\;100\;\times \;\left[1-\left(control\;absorbance/sample\;absorbance\right)\right]$$

### 4.5. Data Analysis

The data were analyzed using the softwares GenStat (VSNi, Hertford, UK) and R [72]. The means and standard errors of difference for the studied traits were obtained through the analysis of restricted maximum likelihood (REML). The variances were analyzed using the Satterthwaite [73] approach to compute broad sense heritability (H^2^) estimates. Correlations among the studied traits were computed according to Holland [74]. For each trait, the least significance mean difference (LSD) was compared for each genotype across sites using Fisher’s test (*p* < 0.05).

The contribution of each genotype of sweet potato storage root to the recommended dietary allowance (RDA) or to the recommended adequate intake (RAI), i.e., the level of nutrients that must be present in the daily diet to meet the requirements of individuals in a given population, was calculated. The genotypes’ grand mean beta-carotene intakes for vitamin A retinol equivalent (µg mg^−1^) RDA/RAI intake were calculated by assuming an intake of 250 g d^−1^ of dry sweet potato flesh. These daily RDA/RAI values for the age group ≤12 months to ≤19 years, including pregnant and breastfeeding women, are 500, 400, 700, 770, and 1300 mg d^−1^ for the age group ≤12 months, 4–8 years, ≥19 years, and pregnant and breastfeeding women, respectively [75,76]. The contribution to the RDA/RAI was calculated by

$$RDA/RAI=\;\frac{\;nutrient\;content\;in\;250\;g}{RDA/RAI\;of\;each\;age\;group}\;\;\times \;100$$

## 5. Conclusions

This study showed consistent variations in the nutraceutical traits and free radical scavenging activities of orange-fleshed sweet potato storage root tubers depending both on the genotype and locality and on their interaction. These variations need to be considered when conducting the varietal selection of genotypes for the introduction of sweet potatoes in the Afar region and any other different agro-ecology where OFSP is not grown. Moreover, this study also demonstrated a high possibility of sweet potato production for storage root yield in arid agro-climate regions with limited production inputs. The genotypes in Aba’ala (arid lowland) had a higher dry matter and beta-carotene content than those in Mekelle-1 and Mekelle-2 (semi-arid highlands).

Ininda, Gloria, and Amelia were the genotypes that provided the higher yield and dry matter content, as well as high levels of starch and beta-carotene; they also showed a high antioxidant power. The mean % RDA/RAI calculated from the daily intake of 250 g (dw) of storage root was high in the same genotypes for vitamin A retinol equivalent: high enough to meet 100% RDA for age groups ≤8 years and more than 50% for the other age groups (≥19 years old and pregnant mothers). These features justify the increased potentials of OFSP in assuaging vitamin A deficiencies. This study demonstrated that sweet potato storage root improvement for yield, dry matter, beta-carotene, starch, and polyphenols is possible in a single genotype through selection. Further investigation is needed on the promising sweet potato genotypes in line with the crop’s wider potential uses.

## Figures and Tables

**Table 1:** Soil chemical and physical properties at the investigated sites.

Parameters	Aba’ala		Mekelle-1		Mekelle-2	
OC (%)	1.90	Moderate	0.48	Low	0.52	Low
Total N (%)	0.14	Low	0.09	Very low	0.10	Very low
Available K (ppm)	121	High	134	High	134	High
Available P (ppm)	38.7	High	26.6	High	26.6	High
Fe (ppm)	15.1	High	11.0	High	11.0	High
Zn (ppm)	6.5	Moderate	5.4	Moderate	5.4	Moderate
Ca (ppm)	25.4	Moderate	29.4	Moderate	29.4	Moderate
CEC (cmol/kg)	38.0	High	41.3	High	41.3	High
EC (ds/m)	0.11	Salt free	0.23	Negligible	0.23	Negligible
pH	6.9	Neutral	7.0	Neutral	7.0	Neutral
Sand (%)	28		18		18	
Silt (%)	54		49		49	
Clay (%)	18		33		33	
Soil type	Silty loam		Silty clay		Silty clay	

OC = organic carbon; CEC = cation exchange capacity; EC = electrical conductivity.

**Table 2:** Two-way ANOVA of nutraceutical traits and yield of sweet potato genotypes at the investigated locations; * = *p* < 0.05.

Parameter	Genotype	Location	Interaction	CV	Residual
Yield	1128.41 *	685.86 *	22.75 *	1.8	10.84
Dry matter	149.90 *	35.50 *	1.54 *	2.0	1.48
Beta-carotene	2.01 *	10.63 *	1.41 *	2.6	0.07
Flavonoids	0.83 *	1.96 *	0.84 *	1.7	0.03
Polyphenols	0.13 *	1.91 *	0.21 *	1.5	0.01
Sucrose	23.14 *	167.03 *	11.09 *	2.6	0.13
Glucose	0.89 *	3.99 *	0.69 *	3.3	0.00
Fructose	2.78 *	4.09 *	1.51 *	1.9	0.00
Starch	605.30 *	1979.09 *	512.06 *	2.1	30.83
Soluble proteins	51.46 *	278.24 *	0.48 *	2.9	0.02
Antiradical activity (ARA %)	219.45 *	142.61 *	37.88 *	1.2	8.92

**Table 3:** Storage root yield and nutraceutical traits (mean ± standard error) of sweet potato genotypes at the three investigated locations.

Locality	Genotype	Y (t/ha)	DM (%)	BC (µg/mg)	FL (µg/mg)	PP (µg/mg)	SU (%)	GL (%)	FR (%)	ST (%)	SP (%)	ARA (%)
Aba’ala	Amelia	6.9 ± 0.1	33.2 ± 1.2	0.73 ± 0.05	0.44 ± 0.03	0.34 ± 0.04	3.86 ± 0.27	0.38 ± 0.07	0.20 ± 0.00	35.53 ± 4.31	2.79 ± 0.13	33.06 ± 4.50
Aba’ala	Gloria	12.7 ± 0.2	34.6 ± 0.1	0.38 ± 0.02	1.39 ± 0.22	1.28 ± 0.02	3.85 ± 0.11	0.19 ± 0.00	0.82 ± 0.05	61.85 ± 5.29	1.46 ± 0.03	47.21 ± 1.55
Aba’ala	Ininda	31.8 ± 0.1	31.0 ± 0. 8	1.34 ± 0.08	2.04 ± 0.07	1.15 ± 0.02	3.60 ± 0.04	0.32 ± 0.03	0.50 ± 0.05	46.42 ± 1.19	1.73 ± 0.18	48.27 ± 2.12
Aba’ala	Kulfo	3.1 ± 0.1	26.3 ± 0.3	1.94 ± 0.07	1.79 ± 0.03	1.05 ± 0.01	4.33 ± 0.25	0.19 ± 0.00	0.40 ± 0.04	69.59 ± 2.50	1.66 ± 0.07	36.10 ± 0.59
Aba’ala	Melinda	3.4 ± 0.7	26.2 ± 0.7	1.97 ± 0.13	1.52 ± 0.19	0.72 ± 0.09	1.64 ± 0.10	0.20 ± 0.00	0.20 ± 0.00	61.15 ± 1.93	2.08 ± 0.09	24.68 ± 5.62
Mekelle-1	Amelia	15.0 ± 0.1	30.9 ± 0. 5	2.39 ± 0.13	1.55 ± 0.05	0.71 ± 0.13	4.43 ± 0.10	0.53 ± 0.03	1.05 ± 0.01	49.97 ± 2.35	0.63 ± 0.05	41.40 ± 3.69
Mekelle-1	Gloria	21.4 ± 0.1	32.3 ± 0.5	1.27 ± 0.07	1.76 ± 0.08	1.10 ± 0.08	7.03 ± 0.10	0.20 ± 0.00	1.21 ± 0.05	78.94 ± 2.96	0.20 ± 0.01	52.38 ± 0.28
Mekelle-1	Ininda	34.9 ± 0.8	28.5 ± 0.2	2.72 ± 0.11	2.82 ± 0.11	1.21 ± 0.11	6.36 ± 0.36	0.47 ± 0.01	0.20 ± 0.00	47.43 ± 1.44	0.44 ± 0.01	50.90 ± 0.41
Mekelle-1	Kulfo	9.2 ± 1.0	24.0 ± 1.5	1.25 ± 0.12	2.09 ± 0.15	1.09 ± 0.12	8.44 ± 0.40	0.19 ± 0.00	0.19 ± 0.00	77.87 ± 3.27	0.80 ± 0.11	64.82 ± 0.55
Mekelle-1	Melinda	11.8 ± 0.3	22.9 ± 1.8	1.38 ± 0.08	1.10 ± 0.05	0.88 ± 0.08	2.81 ± 0.40	0.22 ± 0.01	0.19 ± 0.00	87.49 ± 2.04	1.87 ± 0.13	43.10 ± 0.52
Mekelle-2	Amelia	23.2 ± 0.7	30.5 ± 0.9	3.72 ± 0.25	1.13 ± 0.07	0.32 ± 0.06	8.12 ± 0.55	0.20 ± 0.00	0.20 ± 0.01	41.56 ± 5.27	1.77 ± 0.03	28.67 ± 4.74
Mekelle-2	Gloria	27.9 ± 0.1	32.3 ± 1.2	2.52 ± 0.10	1.03 ± 0.03	0.35 ± 0.01	11.66 ± 0.18	2.48 ± 0.03	3.19 ± 0.05	52.49 ± 2.32	1.47 ± 0.06	30.97 + 0.58
Mekelle-2	Ininda	43.0 ± 0.2	28.7 ± 1.4	3.75 ± 0.22	1.97 ± 0.05	0.35 ± 0.02	13.22 ± 0.09	0.95 ± 0.01	1.94 ± 0.03	40.98 ± 2.29	1.56 ± 0.04	26.17 ± 0.85
Mekelle-2	Kulfo	19.2 ± 1.1	23.5 ± 0.8	1.98 ± 0.04	1.53 ± 0.01	0.66 ± 0.01	11.42 ± 0.37	1.42 ± 0.06	1.94 ± 0.05	47.22 ± 0.76	1.52 ± 0.02	51.26 ± 1.75
Mekelle-2	Melinda	11.8 ± 0.8	23.2 ± 0.5	2.64 ± 0.08	1.07 ± 0.03	0.34 ± 0.01	5.79 ± 0.18	0.85 ± 0.03	0.20 ± 0.00	42.16 ± 2.26	1.81 ± 0.02	25.97 ± 0.77

Y = yield; DM = dry matter; BC = beta-carotene; FL = flavonoids; PP = polyphenols; SU = sucrose; GL = glucose; FR= fructose; ST = starch; SP = soluble proteins; ARA = antiradical activity.

**Table 4:** Antioxidant properties of sweet potato flesh extracts at different concentrations.

Genotypes	200%	100%	50%
Amelia	33.06 ± 4.50	41.40 ± 3.69	28.67 ± 4.74
Gloria	30.97 ± 0.58	52.38 ± 0.28	47.21 ± 1.55
Ininda	26.17 ± 0.85	50.90 ± 0.41	48.27 ± 2.12
Kulfo	51.26 ± 1.75	64.82 ± 0.55	36.10 ± 0.59
Melinda	25.97 ± 0.77	43.10 ± 0.52	24.68 ± 5.62

**Table 5:** Broad sense heritability (H^2^) of nutraceutical traits and storage root yield.

Random Term	H^2^	Standard Error
Yield	0.27	0.10
Dry matter	0.70	0.30
Beta-carotene	0.91	0.01
Flavonoids	0.16	0.01
Polyphenols	0.12	0.00
Sucrose	0.27	0.05
Glucose	0.12	0.00
Fructose	0.22	0.00
Starch	0.69	0.08
Soluble proteins	0.33	0.01
Antiradical activity	0.11	0.09

**Table 6:** Pearson’s correlation coefficient among nutraceutical traits and storage root yield.

	BC	DM	ARA	FL	FR	GL	PP	SP	SU	ST	Y
**BC**	1.00										
**DM**	−0.18	1.00									
**ARA**	−0.58 *	−0.13	1.00								
**FL**	−0.09	−0.09	0.45 *	1.00							
**FR**	0.19	0.26	−0.08	−0.17	1.00						
**GL**	0.32 *	0.00	−0.21	−0.25	0.87 *	1.00					
**PP**	−0.50 *	0.09	0.69 *	0.76 *	−0.26	−0.47 *	1.00				
**SP**	−0.09	−0.07	−0.47 *	−0.71 *	−0.18	−0.01	−0.56 *	1.00			
**SU**	0.66 *	−0.14	−0.13	−0.01	0.68 *	0.68 *	−0.40 *	−0.26	1.00		
**ST**	−0.19	0.02	0.45 *	0.76 *	−0.27	−0.34 *	0.63 *	−0.72 *	−0.16	1.00	
**Y**	0.53 *	0.27 *	−0.08	0.05	0.40 *	0.39 *	−0.11	−0.33 *	0.57 *	0.09 *	1.00

Y = storage root yield; DM = dry matter content; BC = beta-carotene; FL = flavonoids; PP = polyphenols; SU = sucrose; GL = glucose; FR= fructose; ST = starch; SP = soluble proteins; ARA = antiradical activity; * = *p* < 0.05.

**Table 7:** Percentage of recommended dietary allowance/recommended adequate intake for vitamin A supplied by the studied OFSP genotypes.

RDA/RAI (mg d^−1^)		Age Group	Genotypes				
			Amelia	Gloria	Ininda	Kulfo	Melinda
Vitamin A retinol equivalent	500	6–12 months	114	103	130	86	100
	400	4–8 years	143	129	163	108	125
	700	≥19 years	82	73	93	61	71
	770	Pregnant women 30–50 years	74	67	84	56	65
	1300	Lactation 19–50 years	44	40	50	33	38

RDA = recommended dietary allowance; RAI = recommended adequate intake.

## Data Availability

Data are available on reasonable request from the corresponding author.
